# Therapeutic Targeting of Transcription Factors to Control the Cytokine Release Syndrome in COVID-19

**DOI:** 10.3389/fphar.2021.673485

**Published:** 2021-06-07

**Authors:** Clarissa S. Santoso, Zhaorong Li, Jaice T. Rottenberg, Xing Liu, Vivian X. Shen, Juan I. Fuxman Bass

**Affiliations:** ^1^ Department of Biology, Boston University, Boston, MA, United States; ^2^ Bioinformatics Program, Boston University, Boston, MA, United States

**Keywords:** COVID-19, cytokine release syndrome, cytokine storm, drug repurposing, transcriptional regulators, SARS-CoV2, gene regulatory networks

## Abstract

Treatment of the cytokine release syndrome (CRS) has become an important part of rescuing hospitalized COVID-19 patients. Here, we systematically explored the transcriptional regulators of inflammatory cytokines involved in the COVID-19 CRS to identify candidate transcription factors (TFs) for therapeutic targeting using approved drugs. We integrated a resource of TF-cytokine gene interactions with single-cell RNA-seq expression data from bronchoalveolar lavage fluid cells of COVID-19 patients. We found 581 significantly correlated interactions, between 95 TFs and 16 cytokines upregulated in the COVID-19 patients, that may contribute to pathogenesis of the disease. Among these, we identified 19 TFs that are targets of FDA approved drugs. We investigated the potential therapeutic effect of 10 drugs and 25 drugs combinations on inflammatory cytokine production, which revealed two drugs that inhibited cytokine production and numerous combinations that show synergistic efficacy in downregulating cytokine production. Further studies of these candidate repurposable drugs could lead to a therapeutic regimen to treat the CRS in COVID-19 patients.

## Introduction

Coronavirus Disease-2019 (COVID-19), caused by the SARS-CoV-2 betacoronavirus strain, has led to over 170 million confirmed cases and 3.5 million deaths worldwide, since its first reported case in December 2019 (Dong et al., 2020). Most COVID-19 cases are either asymptomatic or cause only mild disease (Gandhi et al., 2020). However, a considerable number of patients develop severe respiratory illnesses manifested in fever and pneumonia, leading to acute respiratory distress syndrome (ARDS) and cytokine release syndrome (CRS) (Mehta et al., 2020). CRS is an acute systemic inflammatory response characterized by the rapid and excessive release of inflammatory cytokines. Uncontrolled CRS results in systemic hyperinflammation and can lead to life-threatening multi-organ failure (Mehta et al., 2020).

There is an urgent need for therapies to treat the CRS in COVID-19 patients. While government agencies and private companies have accelerated procedures to develop and distribute COVID-19 vaccines, it will take a year or longer for the population to be vaccinated. Additionally, a significant portion of the population may not get vaccinated due to reduced compliance and limited access to vaccines, or may not mount a proper protective response (e.g., immunodeficient patients). Furthermore, whether the vaccines generate a long-lasting protective response in all patients is still unknown. Since drug development and approval may take years, drug repurposing of already approved drugs is an efficient approach to identify alternative therapeutic options. At present, three repurposed drugs, remdesivir, dexamethasone, and baricitinib (in combination with remdesivir), have been found to benefit COVID-19 patients in large, controlled, randomized, clinical trials (Beigel et al., 2020; Kalil et al., 2020; Horby et al., 2021). Dexamethasone, which acts as an agonist of the glucocorticoid receptor (GR, also known as NR3C1) transcription factor (TF), is an anti-inflammatory corticosteroid (Cronstein et al., 1992). Indeed, corticosteroids have been shown to suppress CRS (Lee et al., 2014), and NR3C1 has been shown to transcriptionally downregulate many inflammatory cytokines overexpressed in COVID-19 patients, such as CCL2, IL1B, and IL6 (Kadiyala et al., 2016; Sasse et al., 2016). Baricitinib, a non-steroidal anti-inflammatory drug, acts as a janus kinase (JAK) inhibitor (Kalil et al., 2020). The JAK-signal transducers and activators of transcription (STAT) signaling pathway leads to the transcription of inflammatory cytokines, thus inhibition of the JAK-STAT signaling pathway decreases the production of inflammatory cytokines. Despite the efficacy of these drugs in reducing COVID-19 mortality, the effect-size is modest, suggesting the need for additional drugs or combinations to treat the CRS in COVID-19 patients. Although antibodies are well-proven strategies to block cytokine activity, approved antibodies are available for only nine cytokines [DrugBank (Wishart et al., 2018)], specifically TNF and various interleukins (ILs). However, the COVID-19 CRS primarily manifests in overproduction of chemokines (i.e., CCLs and CXCLs) (Liao et al., 2020; Zhou et al., 2020). Thus, as cytokines are highly transcriptionally regulated, there is great potential in exploring other transcriptional regulators of inflammatory cytokines involved in the COVID-19 CRS that can be targeted with approved drugs.

Here, we systematically studied the transcriptional regulators of inflammatory cytokines involved in the COVID-19 CRS to identify candidate TFs for therapeutic targeting using approved drugs. We integrated a resource of empirically identified TF-cytokine gene interactions with single-cell RNA-seq (scRNA-seq) expression data from COVID-19 patients to reveal correlated TF-cytokine gene interactions that may contribute to pathogenesis of the disease. We identified candidate TFs that could be targeted using approved drugs and investigated the potential therapeutic effect of 10 drugs on the expression of cytokines upregulated in COVID-19 patients. We also assayed 25 drug combinations and found numerous combinations that show promising synergistic efficacy in downregulating the expression of inflammatory cytokines. In summary, the present study provides a network-based approach focusing on the transcriptional regulators of inflammatory cytokines to identify candidate repurposable drugs to treat the COVID-19 CRS.

## Results

### Delineation of a COVID-19 Cytokine Gene Regulatory Network

We hypothesized that transcriptional regulators whose expressions are significantly correlated with the expression of cytokines upregulated in COVID-19 patients may play a role in the pathogenesis of the COVID-19 CRS. To identify TF-cytokine pairs correlated in expression, we integrated a published resource of 2,260 empirically tested TF-cytokine gene interactions (CytReg v2) (Santoso et al., 2020) with publicly available scRNA-seq data of bronchoalveolar lavage fluid (BALF) cells from nine COVID-19 patients (GSE145926) (Liao et al., 2020) and four healthy controls (GSE145926 and GSE128033) (Morse et al., 2019; Liao et al., 2020). Unsupervised clustering analysis of the scRNA-seq data revealed distinct clusters of ciliated epithelial cells, secretory epithelial cells, natural killer cells, neutrophils, macrophages, myeloid dendritic cells, plasmacytoid dendritic cells, CD4 T cells, CD8 T cells, B cells, and plasma cells, identified by signature genes (Supplementary Figures S1A,B). For each cell type, we identified cytokines that were significantly (Padj < 0.05) upregulated in the COVID-19 patients compared to healthy controls (Supplementary Table S1), and then determined the TFs in CytReg v2 reported to functionally regulate or bind to the transcriptional control regions of these cytokines. To prioritize TFs that may have a role in the pathogenesis of the COVID-19 CRS, we generated gene regulatory networks for TF-cytokine interactions that are significantly correlated across single cells in each cell type (Supplementary Table S2) in the COVID-19 patient BALF samples.

In total, we identified 581 significantly correlated interactions between 95 TFs and 16 cytokines upregulated in the COVID-19 patients. Strikingly, 567 (97.6%) interactions displayed a positive correlation, suggesting that the cytokine upregulation is primarily mediated through activation by transcriptional activators rather than through de-repression by transcriptional repressors. The transcriptional activation could be a result of activated signaling pathways impinging on the TFs or increased TF expression. Indeed, 89 (93.7%) TFs were significantly upregulated in at least one cell type in the COVID-19 patients, many of which are known to be activated by signaling pathways in inflammation. Consistent with this, TFs in 336 of 395 (85.1%) positively correlated interactions that have a regulatory function reported in CytReg v2 were reported to activate expression of the target cytokine gene in various inflammatory contexts (Santoso et al., 2020). This provides evidence that TFs displaying a positive correlation in the COVID-19 cytokine gene regulatory network can functionally activate expression of the target cytokine gene.

TFs that have widespread interactions across many cell types likely play important roles in regulating the COVID-19 CRS. Notably, IRF2, IRF7, and STAT1, were upregulated and positively correlated with multiple cytokines in all cell types. IRFs and STATs play prominent roles in viral infection by regulating interferon (IFN) production and response pathways and potentiating the expression of antiviral genes including inflammatory cytokine genes (Tamura et al., 2008; Park and Iwasaki, 2020). Indeed, dysregulation of the IFN pathways either by inborn errors or the generation of autoantibodies against type I IFNs has been associated with COVID-19 severity (Bastard et al., 2020; Zhang et al., 2020). However, the robust interferon response observed in many severe COVID-19 patients also likely contributes to the CRS (Israelow et al., 2020; Lee and Shin, 2020; Zhou et al., 2020). Thus, the upregulation of IRF2, IRF7, and STAT, in all cell types may be driving the amplification of IFN response pathways and thereby the overproduction of inflammatory cytokines that contribute to COVID-19 CRS pathogenesis. Consistent with this, inhibiting JAK-STAT signaling with baricitinib significantly improved recovery time and survival rates among patients with severe COVID-19, likely by suppressing the CRS (Kalil et al., 2020; Stebbing et al., 2021).

We next focused on TF hubs, TFs that interact with many overexpressed cytokine genes, since they likely play important roles in COVID-19 CRS pathogenesis. We found that TFs with the highest number of positively correlated interactions were well-known pathogen-activated transcriptional activators, such as REL (29 interactions), STAT1 (29 interactions), IRF7 (28 interactions), and NFKB1 (27 interactions). Additionally, we found that NF-κB family members regulated the most number of unique cytokines (e.g., REL–9 cytokines, RELA–9 cytokines, and NFKB1–8 cytokines). This is consistent with NF-κB being a potent inducer of cytokine production and NF-κB hyperactivation being directly implicated in the CRS observed in severe COVID-19 patients (Hirano and Murakami, 2020). Collectively, these findings support that targeting NF-κB may have therapeutic benefits in controlling the CRS in COVID-19 patients.

**FIGURE 1:**
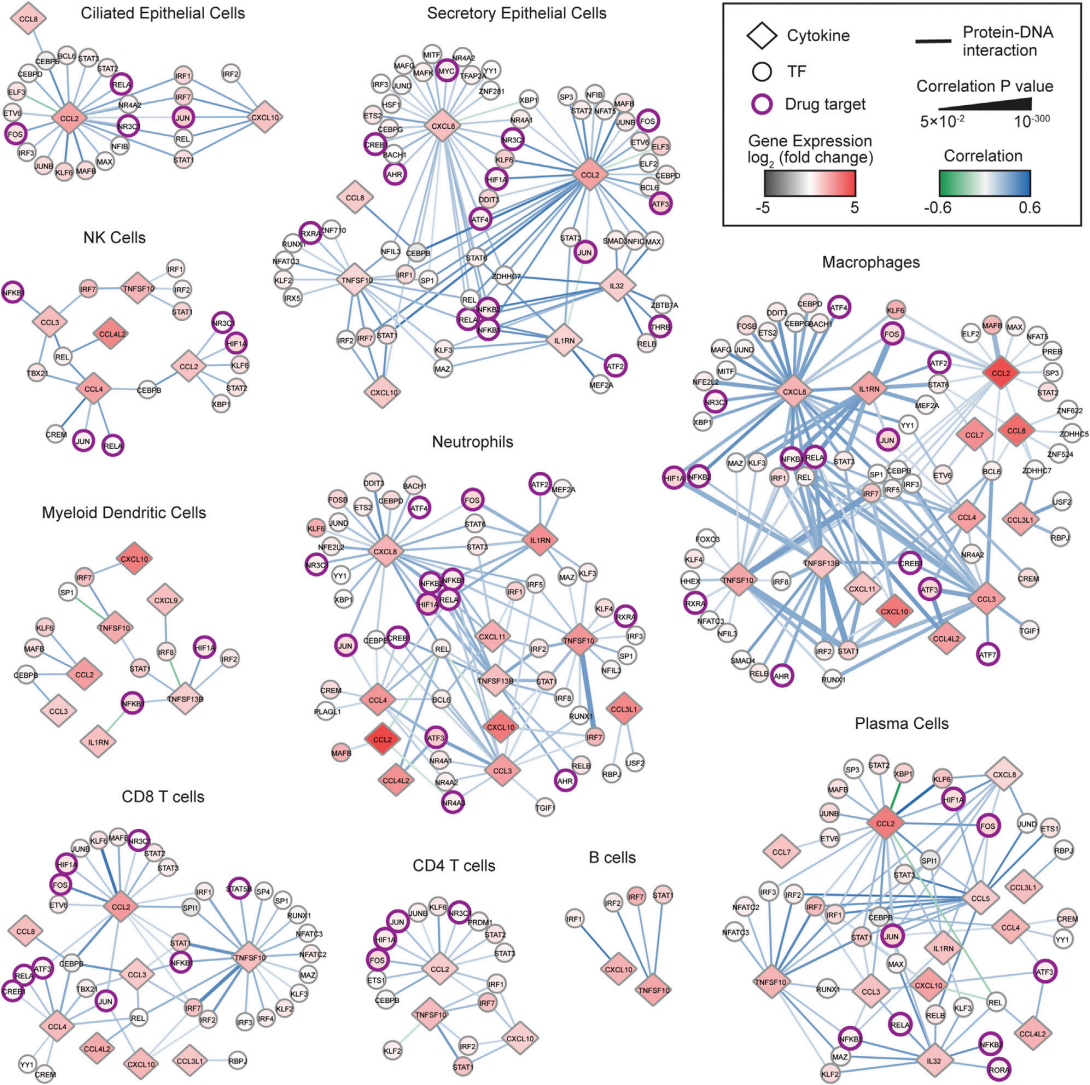
COVID-19 cytokine gene regulatory network. Immune cell sub-networks mapping 581 TF-cytokine gene interactions between 95 TFs and 16 cytokine genes upregulated in BALFs of COVID-19 patients. Networks were restricted to interactions that are significantly correlated (Padj < 0.05) with a Pearson correlation coefficient > 0.1 or < −0.1 in the respective immune cell subtype. Diamonds represent cytokines and circles represent TFs. TFs that are targets of FDA approved drugs are indicated in purple circles. The node color denotes the differential gene expression of TFs and cytokines in the respective immune cell subtype from BALFs of COVID-19 patients compared to healthy controls. The edge color denotes the Pearson correlation coefficient, and the edge thickness is proportional to the correlation adjusted *p* value.

Drug repurposing offers a viable therapeutic approach that can significantly shorten the time to deliver effective treatments to COVID-19 patients. We identified 19 TFs in the networks that are targets of FDA approved drugs (Figure 1 and Supplementary Table S3). Of these, NFKB1, RELA, JUN, FOS, and HIF1A, displayed the highest number of positively correlated interactions and interacted with the most number of unique cytokines. Similar to NF-κB TFs, AP-1 TFs (FOS and JUN) are critical regulators of inflammatory cytokine genes such as CCL2 and IL6 (Akira et al., 1993; Martin et al., 1997), which are expressed at high levels in COVID-19 patients (Blanco-Melo et al., 2020; Huang et al., 2020; Merad and Martin, 2020). Interestingly, CCL2 and IL6 can also induce expression of AP-1 genes and regulate activation of AP-1 proteins (Satoh et al., 1988; Lord et al., 1993; Lin et al., 2012). Therefore, targeting AP-1 has the potential to block these positive feedback loops in addition to limiting the expression of multiple inflammatory cytokines overexpressed in COVID-19 patients.

HIF1A (Hypoxia Inducible Factor 1 Alpha) is a master transcriptional regulator that is activated under hypoxic conditions. Indeed, hypoxia is a primary pathophysiological feature in severe COVID-19 and HIF1A is speculated to contribute largely to the CRS by activating and preventing turnover of immune cells including macrophages and neutrophils, which secrete large amounts of inflammatory cytokines (Walmsley et al., 2005; Nizet and Johnson, 2009; Jahani et al., 2020; Marchetti, 2020). Consistent with this, we found that in seven immune cell types, the expression of HIF1A was positively correlated with the expression of CCL2, CCL5, and CXCL8, which are potent chemoattractants for immature macrophages and neutrophils (Sokol and Luster, 2015), and the expression of TNFSF13B, which promotes cell survival (Lee et al., 2017). These findings suggest that targeting HIF1A could interfere with several processes that contribute to the CRS in COVID-19.

### Targeting TFs to Suppress the Production of Cytokines Involved in the COVID-19 CRS

To reduce the expression of cytokines associated with the COVID-19 CRS, we sought drugs that target the major TF hubs within the network. We prioritized drugs by their status as approved or investigational (i.e., in clinical trials), selectivity, and availability. Based on these criteria, we selected five FDA approved drugs that target the TF hubs (carvedilol–HIF1A, dexamethasone–NR3C1, dimethyl fumarate–RELA, glycyrrhizic acid–NFKB1/2, and sulfasalazine–NF-κB) and one clinical drug (T5224–FOS/JUN), and investigated their ability to downregulate several key cytokines implicated in the COVID-19 CRS (CCL2, CXCL8, and IL6).

**FIGURE 2:**
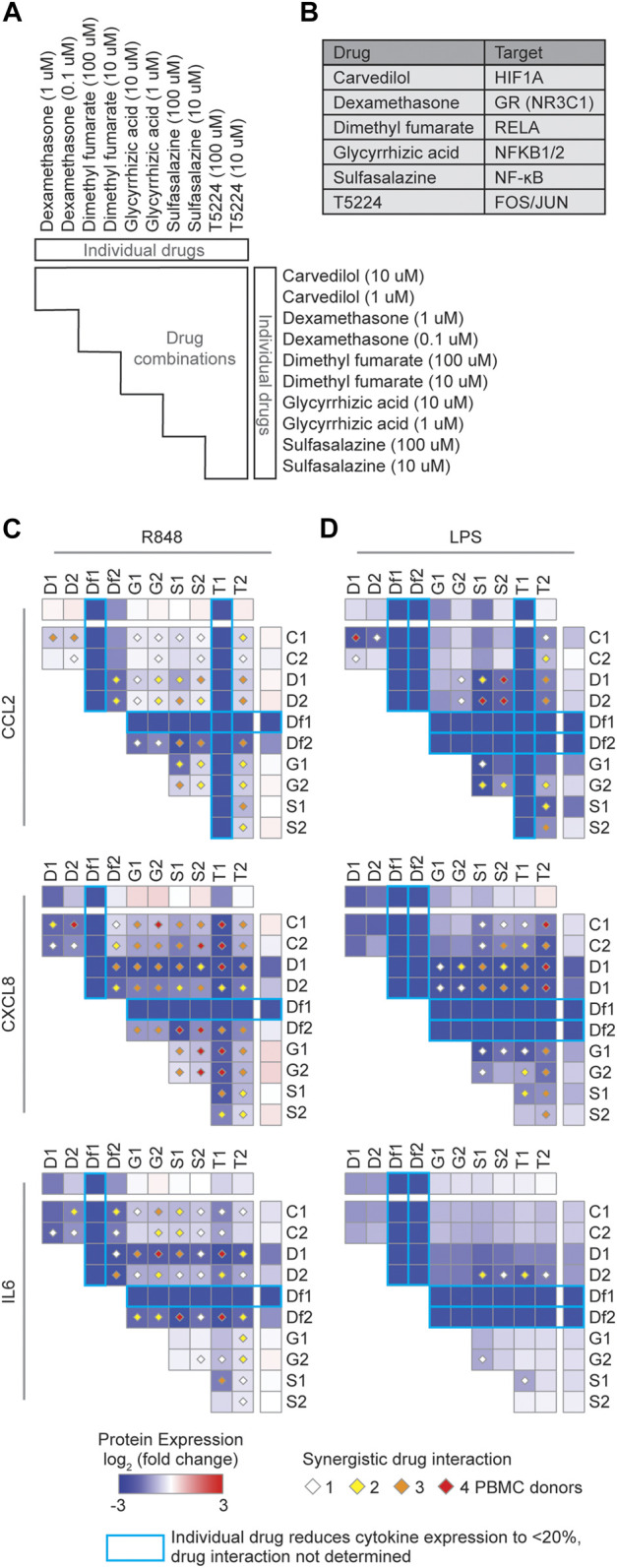
Identification of synergistic drug combinations targeting TF hubs that regulate inflammatory cytokines. **(A)** Schematic of experimental design to test 60 drug combinations. **(B)** Drug TF targets. **(C,D)** Heatmaps showing the average log_2_ (fold change) cytokine production across four PBMC donors treated with the indicated drugs, relative to PBMCs not treated with the indicated drugs, and stimulated with **(C)** R848 or **(D)** LPS. Diamonds indicate synergistic drug interactions, as determined by the coefficient of drug interaction, observed in 1 (white), 2 (yellow), 3 (orange), or all 4 (red) PBMC donors. Blue boxes represent cases wherein the individual drug reduced cytokine expression to less than 20%, and therefore synergistic effects were not evaluated.

We investigated the effect of these drugs, alone and in combination, in peripheral blood mononuclear cells (PBMCs) from four healthy human donors stimulated with R848 or LPS, potent TLR7/TLR8 and TLR4 agonists, respectively (Chow et al., 1999; Hemmi et al., 2002; Jurk et al., 2002). Since TLR7/TLR8 recognize single-stranded RNA from viruses such as SARS-CoV-2 and TLR4, a receptor that recognizes various endogenous and exogenous proteins which was predicted to strongly interact with the SARS-CoV-2 spike glycoprotein (Choudhury and Mukherjee, 2020), activation of these TLR signaling pathways can partially mimic the inflammatory response in COVID-19. We found two drugs, dimethyl fumarate and T5224, that inhibited the production of CCL2, CXCL8, and IL6 (Figures 2C–D). This confirms that targeting TF hubs has the potential to concomitantly limit the production of multiple cytokines upregulated in COVID-19 patients. Additionally, testing all pairwise drug combinations revealed 11 combinations that synergistically reduced the production of at least one cytokine in either stimulated conditions in all PBMC donors (Figures 2C–D). In particular, the combination of dexamethasone with sulfasalazine or T5224 most consistently produced a synergistic effect in reducing cytokine production across the PBMC donors. This may be attributed to these drugs targeting TFs in parallel inflammatory pathways. Collectively, we identified multiple candidate repurposable drugs for the potential treatment of COVID-19 CRS. However, further animal models and clinical trials are required to verify the clinical benefits of these predicted drug candidates.

### Targeting Nuclear Receptors to Suppress the Production of Cytokines Involved in the COVID-19 CRS

**FIGURE 3:**
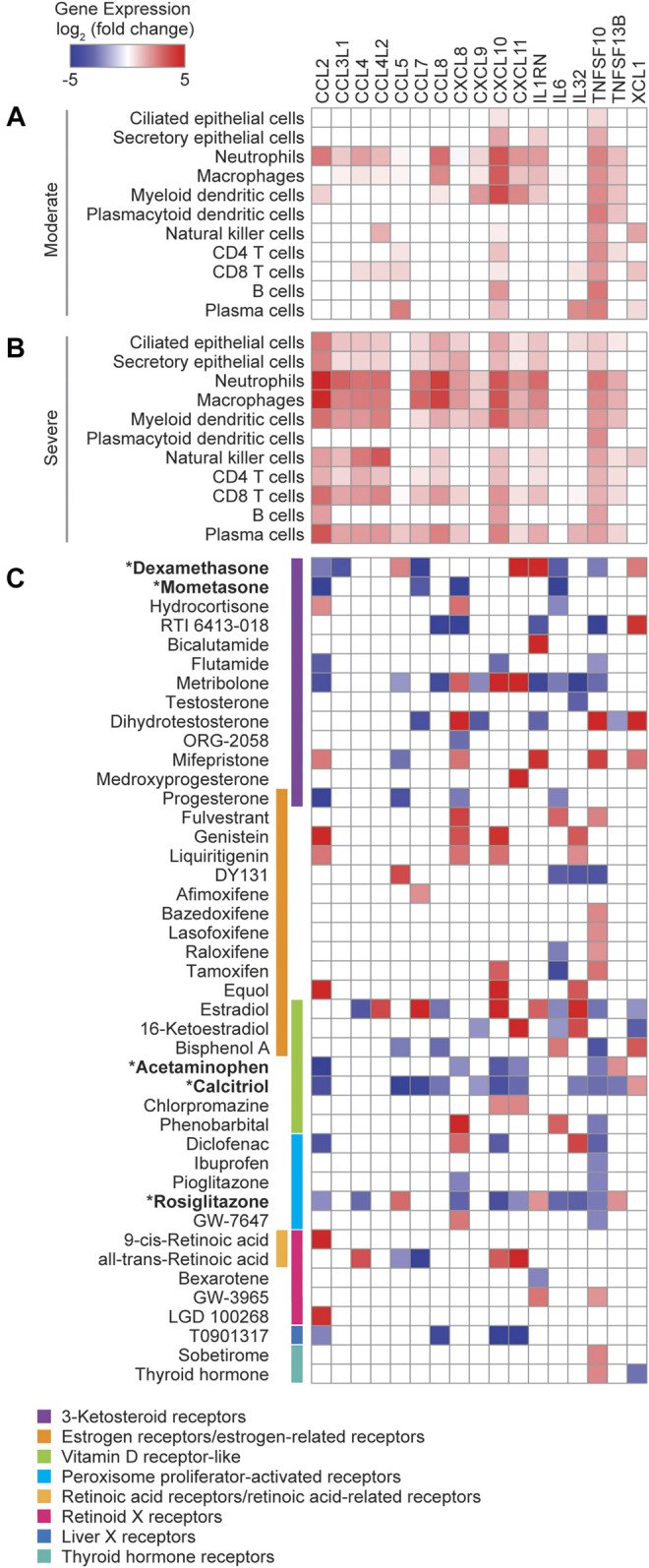
Exploration of repurposable nuclear receptor drugs. **(A,B)** Heatmaps showing the average log_2_ (fold change) cytokine gene expression in the indicated cell types from BALFs of **(A)** moderate and **(B)** severe COVID-19 patients relative to healthy controls. **(C)** Heatmap showing the average log_2_ (fold change) cytokine gene expression in response to treatment with small molecule NR drugs. Data was obtained from the Signaling Pathways Project Transcriptomine resource.

TFs from the nuclear receptor (NR) family present promising therapeutic targets because of the lipophilic nature of their ligands and because numerous FDA approved drugs targeting NRs are currently available. Not surprisingly, only a few NRs were significantly correlated in expression with cytokines overexpressed in the COVID-19 patients (Figure 1), since NRs are ligand-activated TFs and therefore their activities are primarily regulated at the protein level. To explore the therapeutic potential of targeting NRs to reduce the expression of cytokines elevated in COVID-19 patients, we first identified cytokines that were significantly upregulated (Padj < 0.05, fold change ≥2) in the BALFs of moderate (Figure 3A) and severe (Figure 3B) COVID-19 patients compared to healthy controls (Liao et al., 2020). We then analyzed the expression of these cytokines using publicly available transcriptomic data collected from primary human cells and cell lines treated with small molecule NR drugs (Signaling Pathways Project) (Ochsner et al., 2019). We found that, while drugs targeting NRs across many families can modulate the expression of cytokines, drugs targeting members of the 3-ketosteroid, vitamin D, and peroxisome proliferator-activated receptor families, tend to reduce the expression of inflammatory cytokines (Figure 3C).

**FIGURE 4:**
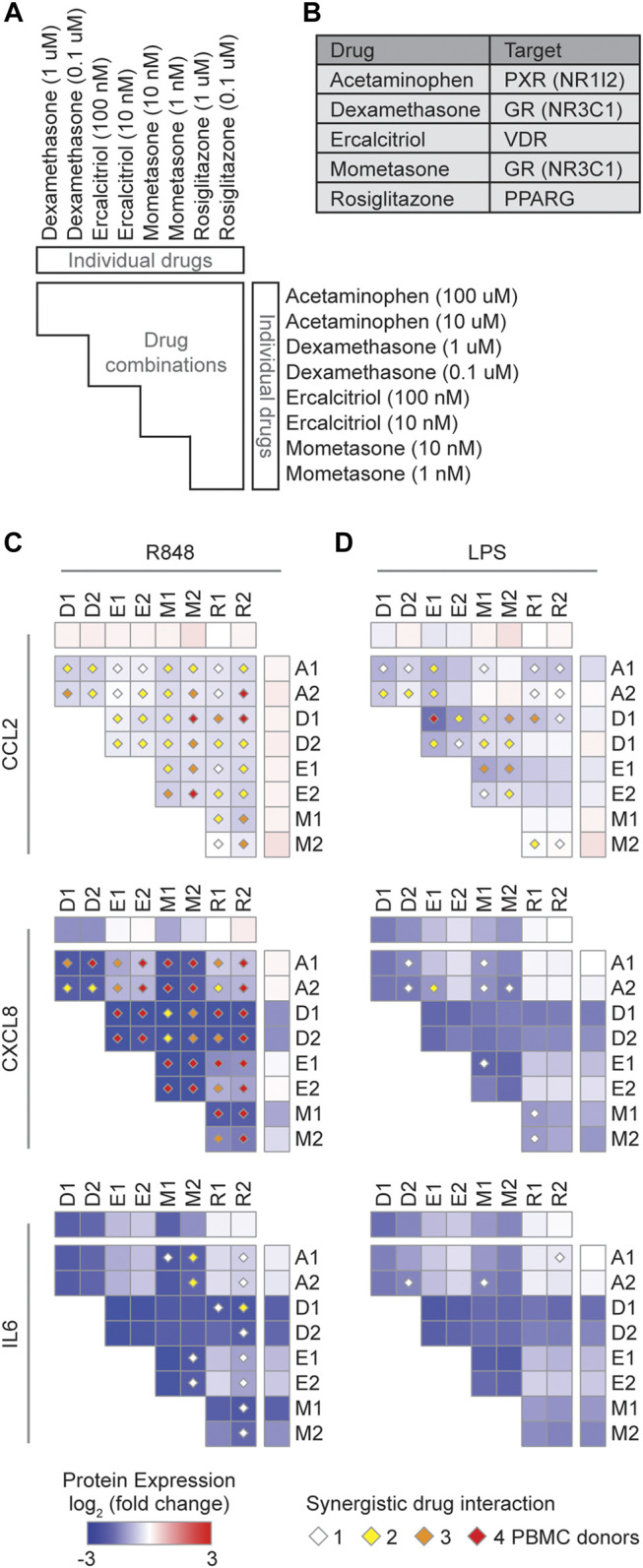
Identification of synergistic drug combinations targeting nuclear receptors that regulate inflammatory cytokines. **(A)** Schematic of experimental design to test 40 drug combinations. **(B)** Drug NR TF targets. **(C,D)** Heatmaps showing the average log_2_ (fold change) cytokine production across four PBMC donors treated with the indicated drugs, relative to PBMCs not treated with the indicated drugs, and stimulated with **(C)** R848 or **(D)** LPS. Diamonds indicate synergistic drug interactions, as determined by the coefficient of drug interaction, observed in 1 (white), 2 (yellow), 3 (orange), or all 4 (red) PBMC donors.

We next investigated the therapeutic potential of five approved NR drugs (acetaminophen, dexamethasone, ercalcitriol, mometasone, and rosiglitazone) that strongly downregulated the expression of multiple cytokines in the expression profiling datasets (Figure 3C), and assayed their effect on CCL2, CXCL8, and IL6, in R848 or LPS stimulated PBMCs (Figures 4A–D). Indeed, TF targets of some of these drugs, for example NR3C1 and VDR, have been reported to directly regulate CCL2, CXCL8, and IL6 expression in other stimulated contexts (Masood et al., 2000; Kadiyala et al., 2016; Sasse et al., 2016). We found that dexamethasone and mometasone, both of which target NR3C1, most potently reduced the production of CXCL8 and IL6 (Figures 4C–D). Additionally, testing all pairwise combinations revealed that all 10 drug combinations either additively or synergistically reduced the production of CCL2 and CXCL8 in all PBMC samples stimulated with R848 (Figure 4C). Generally, across the PBMC samples, combinations of dexamethasone with rosiglitazone and mometasone with ercalcitriol most consistently produced a synergistic effect in reducing cytokine production, while combinations of dexamethasone or mometasone with ercalcitriol most potently reduced cytokine production. Overall, these findings suggest there may be potential therapeutic benefits of repurposing these NR drugs to suppress the CRS in COVID-19 patients. Further studies are required to determine the clinical benefits of these drug candidates.

## Discussion

In the present study, we used a gene regulatory network approach to identify candidate TFs that regulate cytokines overexpressed in COVID-19 patients and evaluated approved drugs targeting these TFs for their ability to downregulate three key cytokines frequently associated with disease severity. We identified two drugs (dimethyl fumarate and T5224) that individually potently suppressed cytokine production, and 25 drug combinations that could synergistically suppress cytokine production. Altogether, these findings provide several promising candidate drugs and targets with potential therapeutic effects for controlling the CRS in COVID-19.

Our network-based approach identified TF hubs that likely regulate many of the cytokines overexpressed in COVID-19 patients. We showed that by targeting these TF hubs, for example targeting RELA with dimethyl fumarate and AP-1 with T5224, we were able to concomitantly inhibit the production of multiple cytokines. Moreover, targeting TF hubs may also interfere with positive feedback and feedforward loops of cytokine production that lead to the CRS (Hirano and Murakami, 2020). It would also be interesting to explore targeting non-hub TFs that regulate a key cytokine responsible for driving these loops, as the effects could me more specific with less side effects.

Combination therapies have the potential to increase drug efficacy and reduce side effects, and have thus become a routine strategy in the treatment of diseases (Sun et al., 2016). In particular, synergistic combinations allow the use of lower doses to achieve the same effect as the individual drugs, which may reduce adverse reactions. Notably, nearly all drugs we tested achieved a similar or stronger suppression of cytokine production when used at a 10-fold lower dose in combination than when used individually. This includes the combination of dexamethasone with ercalcitriol (active metabolite of vitamin D). Thus, in the debate of whether vitamin D supplementation has beneficial effects in the treatment of COVID-19, at least from the perspective of treating the CRS, vitamin D may enhance the anti-inflammatory effects of dexamethasone.

Aside from having well-known anti-inflammatory properties, some of the drugs tested also have reported antiviral properties against SARS-CoV-2. Dimethyl fumarate, mometasone, calcitriol, and sulfasalazine, potently inhibited SARS-CoV-2 replication *in vitro* in Vero E6 cells (Han et al., 2020; Matsuyama et al., 2020; Mok et al., 2020; Olagnier et al., 2020). The antiviral activities of Glycyrrhizin have been extensively studied in the context of other human viruses and most notably, the drug was found to potently suppress replication of two clinical isolates of SARS-associated coronavirus in Vero cells (Cinatl et al., 2003), and preliminarily, neutralize SARS-CoV-2 by inhibiting the viral main protease (van de Sand et al., 2021). Further, Carvedilol and Acetaminophen have been reported to decrease the expression of ACE2 and serine protease TMPRSS2, respectively (Zarubin et al., 2020; Skayem and Ayoub, 2020), both of which are required for SARS-CoV-2 entry into cells (Hoffmann et al., 2020). Hence, drug combinations that simultaneously exert both anti-inflammatory and antiviral effects against SARS-CoV-2 may have the greatest potential to be effective in treating COVID-19.

There is growing evidence that certain food supplements may have therapeutic benefits in COVID-19. Numerous small-scale studies have found that patients with sufficient vitamin D levels are less likely to have life-threatening complications (Grant, 2020; Jain et al., 2020; Meltzer et al., 2020). Additionally, glycyrrhizic acid, a frequent component in traditional Chinese medicines and the main constituent in licorice, has been reported to have anti-inflammatory properties, by antagonizing TLR4 (Bailly and Vergoten, 2020; Murck, 2020), and broad antiviral activities (Cinatl et al., 2003). Other foods are also known to inhibit inflammatory mediators, for example curcumin, a substance in turmeric that gives curry its distinct flavor and yellow color, inhibits numerous TFs including NF-κB, AP-1, and HIF1A, and has potent anti-inflammatory properties (Singh and Aggarwal, 1995; Bae et al., 2006). Hence, a study of the association between food intake and the severity of COVID-19 symptoms and outcomes may shed light into differences in severity and mortality between countries (Samaddar et al., 2020).

In summary, our approach of targeting transcriptional regulators of cytokines associated with the CRS provides candidate drugs and targets to treat COVID-19. However, additional research is needed to determine whether these combinations elicit the same immunomodulatory response in the context of SARS-CoV-2 infection. As more scRNA-seq data from COVID-19 patients become available (Ren et al., 2021), analyzing additional datasets could validate our findings. Importantly, although all the drugs investigated in this study are FDA approved, careful evaluation of the efficacy, safety, and risk-benefit balance of these drugs in animal models and COVID-19 patients is necessary as outcomes of drug interactions could drastically differ between *in vitro*, *in vivo*, and clinical trials. Nonetheless, the candidate drugs show promise for further investigation in downregulating the CRS in COVID-19 patients. More broadly, the findings reported here may also be applicable to CRS resulting from other viral infections, bacterial infections, sepsis, and CAR-T therapies.

## Methods

### ScRNA-Seq Data Processing

We utilized BALF samples analyzed in Liao et al. (Liao et al., 2020) and searched the GEO database to download the scRNA-seq datasets. The scRNA-seq datasets were downloaded from two GEO repositories: nine COVID-19 patient samples and three healthy control BALF samples were obtained from GSE145926 (Liao et al., 2020), and another healthy control BALF sample was obtained from GSE128033 (Morse et al., 2019). For all datasets, we used STARsolo (v 2.7.3) (Dobin et al., 2013) to align reads to the human GRCh38 genome and quantify read counts to determine gene expression. We used Scrubblet (Wolock et al., 2019) to detect and remove doublets, and then filtered the remaining data to only retain cells with 1,000–50,000 UMI counts, 500–7,500 genes, and less than 25% mitochondrial reads. A total of 72,433 cells remaining were used for all subsequent analysis. Finally, the data was normalized using the regularized negative binomial regression method (Hafemeister and Satija, 2019) and batch effect was removed using the Canonical Correlation Analysis method (Butler et al., 2018).

Cell clustering was performed using Seurat (v3.1.4) (Butler et al., 2018) and cell type classifications were obtained using SingleR (Aran et al., 2019), and then validated with canonical immune cell type markers. The following markers were used to identify cell types: ciliated epithelial cells: *TUBB4B* and *TPPP3*; secretory epithelial cells: *SCGB3A1* and *SCGB1A1*; neutrophils: *S100A8*, *S100A9* and *FCN1*; macrophages: *APOE*, *C1QA*, and *C1QB*; myeloid dendritic cells: *FCER1A* and *CD1C*; plasmacytoid dendritic cells: *TCF4* and *TNFRSF21*; mast cells: *AREG*, *TPSB2* and *TPSAB1*; NK cells: *GNLY*, *PRF1*, *NKG7* and the absence of the general T cell markers; T cells: *CD3D, CD3G, CD4E, CD4* (CD4 T cells only), and CD8 (CD8 T cells only); B cells: *CD79A*, *CD79B*, and *MS4A1*; plasma cells: *IGHG1*, *IGHG2*, and *IGHG4*.

### Differential Gene Expression Analysis

Differential gene expression analysis, between BALF cells from COVID-19 patients and healthy controls, was performed using a Wilcoxon test, and the *p* values were adjusted by false discovery rate correction using the Benjamini-Hochberg method.

### Correlation Analysis

Correlation coefficients between TFs and cytokines in BALF cells from COVID-19 patients were determined using the Pearson correlation method, and the *p* values were adjusted by false discovery rate correction using the Benjamini-Hochberg method. The correlation analyses were restricted to cells with reads for both the TF and the cytokine, to limit noise and over-estimation of the correlation due to cells with zero reads for either the TF or the cytokine or both, and cell types with more than 10% of cells expressing both the TF and the cytokine. Correlations between TFs and cytokines were determined per cell type.

### Signaling Pathways Project Data Acquisition and Processing

A list of cytokines that were differentially expressed and upregulated in BALFs of COVID-19 patients compared to healthy controls was submitted to the Signaling Pathways Project Ominer web tool (Ochsner et al., 2019) on July 25, 2020. The search criteria included Omics Category: Transcriptomics, Module Category: Nuclear receptors–all families, Biosample Category: Human–all physiological systems, FDR Significance Cut-off: 0.05. The search results were downloaded as a table reporting the fold change for cytokine gene expression in experimental *vs*. control conditions. Only experiments involving small molecule NR drugs were further explored in our analysis. If there were multiple experiments for a drug-cytokine interaction, only interactions wherein at least 80% of the experiments resulted in cytokine gene expression changing in the same direction were included in our analysis. Finally, for each drug-cytokine interaction, we calculated the median fold change in cytokine gene expression across the experiments and depicted the data in a heatmap.

### Peripheral Blood Mononuclear Cells Purification and Drug Treatment

Peripheral blood mononuclear cells (PBMCs) were isolated from de-identified human leukapheresis-processed blood (New York Biologics, Inc.) by centrifugation through Lymphoprep (Stem Cell Technologies.) density gradient. PBMCs were washed in PBS, resuspended in red blood cell lysis solution for 5 min, and washed three more times in PBS. Purified PBMCs were cultured in RPMI supplemented with 10% FBS and 1% Antibiotic-Antimycotic (100X) and plated in 96-well plates at a density of 1 × 10^6^ cells/ml and 0.1 ml/well. Purified PBMCs were immediately treated with the different drugs or frozen in RPMI supplemented with 40% FBS, 10% DMSO, and 1% Antibiotic-Antimycotic (100X). Frozen PBMCs were rapidly thawed in a 37°C water bath, washed three times in warm RPMI supplemented with 10% FBS and 1% Antibiotic-Antimycotic (100X), and rested for 1 h at 37°C with 5% CO_2_ before drug treatment. The data in Figure 2 were obtained from frozen PBMCs and the data in Figure 4 were obtained from fresh PBMCs. Fresh and frozen PBMCs were tested by trypan blue viability assay and only used when the viability was >90%. PBMCs were pretreated with Acetaminophen (MiliporeSigma), Dexamethasone (MiliporeSigma), Ercalcitriol (Tocris), Mometasone (Tocris), or Rosiglitazone (Tocris), at the various concentrations for 30 min, and then stimulated with R848 (1 µM) or LPS (100 ng/ml) for 20 h. The supernatants were collected and the amounts of CCL2, CXCL8, and IL6, were quantified by ELISA. Fresh and frozen PBMCs, from PBMC donor 1, untreated and treated with the various drugs exhibited similar cytokine levels (data not shown). Each experimental condition was tested in four biological replicates or PBMC donors, and each experimental condition was performed in two technical replicates for each PBMC donor. The average of the replicates was used to determine cytokine expression.

### Measurement of Cytokine Production

The amount of cytokines (CCL2, CXCL8, and Il6) in treated PBMC supernatants were quantified by ELISA using the ELISA MAX Deluxe Set Human CCL2 (Biolegend), ELISA MAX Deluxe Set Human IL8 (Biolegend), and ELISA MAX Deluxe Set Human IL6 (Biolegend) kits according to the manufacturer’s protocol.

### Calculation of Coefficient of Drug Interaction

To determine drug interactions (i.e., additive, synergistic, or antagonistic), we calculated the coefficient of drug interaction (CDI) using the formula CDI = AB/(A × B), where AB is the ratio of the combination to the control, and A or B is the ratio of the single drug to the control. Thus, a CDI = 1.0 indicates an additive interaction, a CDI < 1.0 indicates a synergistic interaction, and a CDI > 1.0 indicates an antagonistic interaction. We applied more conservative thresholds to determine the drug interaction such that a CDI = 0.7–1.3 indicates an additive interaction, CDI < 0.7 indicates a synergistic interaction, and CDI > 1.3 indicates an antagonistic interaction.

## Significance

The cytokine release syndrome (CRS) contributes largely to the morbidity and mortality in COVID-19 patients. While antibodies are well-proven strategies to block cytokine activity, approved antibodies are available for only a few cytokines. Targeting the transcriptional regulators of cytokines involved in the CRS provides an alternative strategy that also allows for the concomitant downregulation of multiple cytokines. In the present study, we explored drugs that target transcriptional factors of cytokine genes overexpressed in COVID-19 patients. We identified two drugs that individually potently suppressed cytokine production, and 25 drug combinations that could synergistically suppress cytokine production. Altogether, our findings provide several promising candidate drugs and targets with potential therapeutic effects for controlling the CRS in COVID-19.

## Data Availability

The original contributions presented in the study are included in the article/Supplementary Material, further inquiries can be directed to the corresponding author.

## References

[B1] Akira S. Taga T. Kishimoto T. (1993). Interleukin-6 in Biology and Medicine. Adv. Immunol. 54, 1–78. 10.1016/s0065-2776(08)60532-5 8379461

[B3] Aran D. Looney A. P. Liu L. Wu E. Fong V. Hsu A. (2019). Reference-based Analysis of Lung Single-Cell Sequencing Reveals a Transitional Profibrotic Macrophage. Nat. Immunol. 20 (2), 163–172. 10.1038/s41590-018-0276-y 30643263 PMC6340744

[B4] Bae M. K. Kim S. H. Jeong J. W. Lee Y. M. Kim H. S. Kim S. R. (2006). Curcumin Inhibits Hypoxia-Induced Angiogenesis via Down-Regulation of HIF-1. Oncol. Rep. 15 (6), 1557–1562. 16685395

[B5] Bailly C. Vergoten G. (2020). Glycyrrhizin: An Alternative Drug for the Treatment of COVID-19 Infection and the Associated Respiratory Syndrome? Pharmacol. Ther. 214, 107618. 10.1016/j.pharmthera.2020.107618 32592716 PMC7311916

[B6] Bastard P. Rosen L. B. Zhang Q. Michailidis E. Hoffmann H. H. Zhang Y. (2020). Autoantibodies against Type I IFNs in Patients with Life-Threatening COVID-19. Science 370 (6515). 10.1126/science.abd4585 PMC7857397 32972996

[B7] Beigel J. H. Tomashek K. M. Dodd L. E. Mehta A. K. Zingman B. S. Kalil A. C. (2020). Remdesivir for the Treatment of Covid-19 - Final Report. N. Engl. J. Med. 383 (19), 1813–1826. 10.1056/NEJMoa2007764 32445440 PMC7262788

[B8] Blanco-Melo D. Nilsson-Payant B. E. Liu W.-C. Uhl S. Hoagland D. Møller R. (2020). Imbalanced Host Response to SARS-CoV-2 Drives Development of COVID-19. Cell 181 (5), 1036–1045. 10.1016/j.cell.2020.04.026 32416070 PMC7227586

[B9] Butler A. Hoffman P. Smibert P. Papalexi E. Satija R. (2018). Integrating Single-Cell Transcriptomic Data across Different Conditions, Technologies, and Species. Nat. Biotechnol. 36 (5), 411–420. 10.1038/nbt.4096 29608179 PMC6700744

[B10] Choudhury A. Mukherjee S. (2020). In Silico studies on the Comparative Characterization of the Interactions of SARS‐CoV‐2 Spike Glycoprotein with ACE‐2 Receptor Homologs and Human TLRs. J. Med. Virol. 92 (10), 2105–2113. 10.1002/jmv.25987 32383269 PMC7267663

[B11] Chow J. C. Young D. W. Golenbock D. T. Christ W. J. Gusovsky F. (1999). Toll-like Receptor-4 Mediates Lipopolysaccharide-Induced Signal Transduction. J. Biol. Chem. 274 (16), 10689–10692. 10.1074/jbc.274.16.10689 10196138

[B12] Cinatl J. Morgenstern B. Bauer G. Chandra P. Rabenau H. Doerr H. (2003). Glycyrrhizin, an Active Component of Liquorice Roots, and Replication of SARS-Associated Coronavirus. The Lancet 361 (9374), 2045–2046. 10.1016/s0140-6736(03)13615-x PMC7112442 12814717

[B13] Cronstein B. N. Kimmel S. C. Levin R. I. Martiniuk F. Weissmann G. (1992). A Mechanism for the Antiinflammatory Effects of Corticosteroids: the Glucocorticoid Receptor Regulates Leukocyte Adhesion to Endothelial Cells and Expression of Endothelial-Leukocyte Adhesion Molecule 1 and Intercellular Adhesion Molecule 1. Proc. Natl. Acad. Sci. 89 (21), 9991–9995. 10.1073/pnas.89.21.9991 1279685 PMC50263

[B14] Dobin A. Davis C. A. Schlesinger F. Drenkow J. Zaleski C. Jha S. (2013). STAR: Ultrafast Universal RNA-Seq Aligner. Bioinformatics 29 (1), 15–21. 10.1093/bioinformatics/bts635 23104886 PMC3530905

[B15] Dong E. Du H. Gardner L. (2020). An Interactive Web-Based Dashboard to Track COVID-19 in Real Time. Lancet Infect. Dis. 20 (5), 533–534. 10.1016/s1473-3099(20)30120-1 32087114 PMC7159018

[B16] Gandhi R. T. Lynch J. B. Del Rio C. (2020). Mild or Moderate Covid-19. N. Engl. J. Med. 383 (18), 1757–1766. 10.1056/nejmcp2009249 32329974

[B17] Grant W. B. (2020). Evidence that Vitamin D Supplementation Could Reduce Risk of Influenza and COVID-19 Infections and Deaths. Nutrients 12 (4). 10.3390/nu12061620 PMC7231123 32252338

[B19] Hafemeister C. Satija R. (2019). Normalization and Variance Stabilization of Single-Cell RNA-Seq Data Using Regularized Negative Binomial Regression. Genome Biol. 20 (1), 296. 10.1186/s13059-019-1874-1 31870423 PMC6927181

[B46] Han N. Hwang W. Tzelepis K. Schmerer P. Yankova E. MacMahon M. (2020). Identification of SARS-CoV-2 Induced Pathways Reveal Drug Repurposing Strategies. BioRxiv. 10.1101/2020.08.24.265496 PMC8245040 34193418

[B20] Hemmi H. Kaisho T. Takeuchi O. Sato S. Sanjo H. Hoshino K. (2002). Small Anti-viral Compounds Activate Immune Cells via the TLR7 MyD88-dependent Signaling Pathway. Nat. Immunol. 3 (2), 196–200. 10.1038/ni758 11812998

[B21] Hirano T. Murakami M. (2020). COVID-19: A New Virus, but a Familiar Receptor and Cytokine Release Syndrome. Immunity 52 (5), 731–733. 10.1016/j.immuni.2020.04.003 32325025 PMC7175868

[B22] Hoffmann M. Kleine-Weber H. Schroeder S. Krüger N. Herrler T. Erichsen S. (2020). SARS-CoV-2 Cell Entry Depends on ACE2 and TMPRSS2 and Is Blocked by a Clinically Proven Protease Inhibitor. Cell 181 (2), 271–280. 10.1016/j.cell.2020.02.052e278 32142651 PMC7102627

[B18] Horby P. Lim W. S. Emberson J. R. Mafham M. Bell J. L. Linsell L. RECOVERY Collaborative Group (2021). Dexamethasone in Hospitalized Patients with Covid-19. N. Engl. J. Med. 384 (8), 693–704. 32678530 10.1056/NEJMoa2021436 PMC7383595

[B23] Huang C. Wang Y. Li X. Ren L. Zhao J. Hu Y. (2020). Clinical Features of Patients Infected with 2019 Novel Coronavirus in Wuhan, China. The Lancet 395 (10223), 497–506. 10.1016/s0140-6736(20)30183-5 PMC7159299 31986264

[B24] Israelow B. Song E. Mao T. Lu P. Meir A. Liu F. (2020). Mouse Model of SARS-CoV-2 Reveals Inflammatory Role of Type I Interferon Signaling. J. Exp. Med. 217 (12), e20201241. 10.1084/jem.20201241 32750141 PMC7401025

[B25] Jahani M. Dokaneheifard S. Mansouri K. (2020). Hypoxia: A Key Feature of COVID-19 Launching Activation of HIF-1 and Cytokine Storm. J. Inflamm. (Lond) 17 (1), 33. 10.1186/s12950-020-00263-3 33139969 PMC7594974

[B26] Jain A. Chaurasia R. Sengar N. S. Singh M. Mahor S. Narain S. (2020). Analysis of Vitamin D Level Among Asymptomatic and Critically Ill COVID-19 Patients and its Correlation with Inflammatory Markers. Sci. Rep. 10 (1), 20191. 10.1038/s41598-020-77093-z 33214648 PMC7677378

[B27] Jurk M. Heil F. Vollmer J. Schetter C. Krieg A. M. Wagner H. (2002). Human TLR7 or TLR8 Independently Confer Responsiveness to the Antiviral Compound R-848. Nat. Immunol. 3 (6), 499. 10.1038/ni0602-499 12032557

[B28] Kadiyala V. Sasse S. K. Altonsy M. O. Berman R. Chu H. W. Phang T. L. (2016). Cistrome-based Cooperation between Airway Epithelial Glucocorticoid Receptor and NF-Κb Orchestrates Anti-inflammatory Effects. J. Biol. Chem. 291 (24), 12673–12687. 10.1074/jbc.m116.721217 27076634 PMC4933445

[B29] Kalil A. C. Patterson T. F. Mehta A. K. Tomashek K. M. Wolfe C. R. Ghazaryan V. (2020). Baricitinib Plus Remdesivir for Hospitalized Adults with Covid-19. N. Engl. J. Med. 384 (9), 795–807. 10.1056/NEJMoa2031994 33306283 PMC7745180

[B30] Lee D. W. Gardner R. Porter D. L. Louis C. U. Ahmed N. Jensen M. (2014). Current Concepts in the Diagnosis and Management of Cytokine Release Syndrome. Blood 124 (2), 188–195. 10.1182/blood-2014-05-552729 24876563 PMC4093680

[B31] Lee J. S. Shin E.-C. (2020). The Type I Interferon Response in COVID-19: Implications for Treatment. Nat. Rev. Immunol. 20 (10), 585–586. 10.1038/s41577-020-00429-3 32788708 PMC8824445

[B32] Lee J. W. Lee J. Um S. H. Moon E. Y. (2017). Synovial Cell Death Is Regulated by TNF-Alpha-Induced Expression of B-Cell Activating Factor through an ERK-dependent Increase in Hypoxia-Inducible Factor-1alpha. Cell Death Dis 8 (4), e2727. 10.1038/cddis.2017.26 28383556 PMC5477592

[B33] Liao M. Liu Y. Yuan J. Wen Y. Xu G. Zhao J. (2020). Single-cell Landscape of Bronchoalveolar Immune Cells in Patients with COVID-19. Nat. Med. 26 (6), 842–844. 10.1038/s41591-020-0901-9 32398875

[B34] Lin Y. M. Hsu C. J. Liao Y. Y. Chou M. C. Tang C. H. (2012). The CCL2/CCR2 axis Enhances Vascular Cell Adhesion Molecule-1 Expression in Human Synovial Fibroblasts. PLoS One 7 (11), e49999. 10.1371/journal.pone.0049999 23185512 PMC3503714

[B35] Lord K. A. Abdollahi A. Hoffman-Liebermann B. Liebermann D. A. (1993). Proto-oncogenes of the Fos/jun Family of Transcription Factors Are Positive Regulators of Myeloid Differentiation. Mol. Cel. Biol. 13 (2), 841–851. 10.1128/mcb.13.2.841 PMC358967 8423806

[B36] Marchetti M. (2020). COVID-19-driven Endothelial Damage: Complement, HIF-1, and ABL2 Are Potential Pathways of Damage and Targets for Cure. Ann. Hematol. 99 (8), 1701–1707. 10.1007/s00277-020-04138-8 32583086 PMC7312112

[B37] Martin T. Cardarelli P. M. Parry G. C. N. Felts K. A. Cobb R. R. (1997). Cytokine Induction of Monocyte Chemoattractant Protein-1 Gene Expression in Human Endothelial Cells Depends on the Cooperative Action of NF-Χb and AP-1. Eur. J. Immunol. 27 (5), 1091–1097. 10.1002/eji.1830270508 9174597

[B38] Masood R. Nagpal S. Zheng T. Cai J. Tulpule A. Smith D. L. (2000). Kaposi Sarcoma Is a Therapeutic Target for Vitamin D3receptor Agonist. Blood 96 (9), 3188–3194. 10.1182/blood.v96.9.3188.h8003188_3188_3194 11050002

[B39] Matsuyama S. Kawase M. Nao N. Shirato K. Ujike M. Kamitani W. (2020). The Inhaled Steroid Ciclesonide Blocks SARS-CoV-2 RNA Replication by Targeting the Viral Replication-Transcription Complex in Cultured Cells. J. Virol. 95 (1). 10.1128/jvi.01648-20 PMC7737752 33055254

[B40] Mehta P. McAuley D. F. Brown M. Sanchez E. Tattersall R. S. Manson J. J. (2020). COVID-19: Consider Cytokine Storm Syndromes and Immunosuppression. The Lancet 395 (10229), 1033–1034. 10.1016/s0140-6736(20)30628-0 PMC7270045 32192578

[B41] Meltzer D. O. Best T. J. Zhang H. Vokes T. Arora V. Solway J. (2020). Association of Vitamin D Status and Other Clinical Characteristics with COVID-19 Test Results. JAMA Netw. Open 3 (9), e2019722. 10.1001/jamanetworkopen.2020.19722 32880651 PMC7489852

[B42] Merad M. Martin J. C. (2020). Pathological Inflammation in Patients with COVID-19: a Key Role for Monocytes and Macrophages. Nat. Rev. Immunol. 20 (6), 355–362. 10.1038/s41577-020-0331-4 32376901 PMC7201395

[B43] Mok C. K. Ahidjo B. A. Ng Y. L. Loe M. W. C. Ahidjo B. A. Hua Lee R. C. (2020). Tze Minn Mak, Cui Lin, Raymond Lin, Paul Tambyah, JiaGang Deng, Justin Jang Hann ChuCalcitriol, the Active Form of Vitamin D, Is a Promising Candidate for COVID-19 Prophylaxis. BioRxiv. 10.1101/2020.06.21.162396

[B44] Morse C. Tabib T. Sembrat J. Buschur K. L. Bittar H. T. Valenzi E. (2019). Proliferating SPP1/MERTK-Expressing Macrophages in Idiopathic Pulmonary Fibrosis. Eur. Respir. J. 54 (2). 10.1183/13993003.02441-2018 PMC8025672 31221805

[B45] Murck H. (2020). Symptomatic Protective Action of Glycyrrhizin (Licorice) in COVID-19 Infection? Front. Immunol. 11, 1239. 10.3389/fimmu.2020.01239 32574273 PMC7270278

[B47] Nizet V. Johnson R. S. (2009). Interdependence of Hypoxic and Innate Immune Responses. Nat. Rev. Immunol. 9 (9), 609–617. 10.1038/nri2607 19704417 PMC4343208

[B48] Ochsner S. A. Abraham D. Martin K. Ding W. McOwiti A. Kankanamge W. (2019). The Signaling Pathways Project, an Integrated 'omics Knowledgebase for Mammalian Cellular Signaling Pathways. Sci. Data 6 (1), 252. 10.1038/s41597-019-0193-4 31672983 PMC6823428

[B49] Olagnier D. Farahani E. Thyrsted J. Blay-Cadanet J. Herengt A. Idorn M. (2020). SARS-CoV2-mediated Suppression of NRF2-Signaling Reveals Potent Antiviral and Anti-inflammatory Activity of 4-Octyl-Itaconate and Dimethyl Fumarate. Nat. Commun. 11 (1), 4938. 10.1038/s41467-020-19363-y 33009401 PMC7532469

[B50] Park A. Iwasaki A. (2020). Type I and Type III Interferons - Induction, Signaling, Evasion, and Application to Combat COVID-19. Cell Host *& *Microbe 27 (6), 870–878. 10.1016/j.chom.2020.05.008 32464097 PMC7255347

[B51] Ren X. Wen W. Fan X. Hou W. Su B. Cai P. (2021). COVID-19 Immune Features Revealed by a Large-Scale Single-Cell Transcriptome Atlas. Cell 184 (7), 1895–e19. 10.1016/j.cell.2021.01.053 33657410 PMC7857060

[B52] Samaddar A. Gadepalli R. Nag V. L. Misra S. (2020). The Enigma of Low COVID-19 Fatality Rate in India. Front. Genet. 11, 854. 10.3389/fgene.2020.00854 32849833 PMC7399343

[B53] Santoso C. S. Li Z. Lal S. Yuan S. Gan K. A. Agosto L. M. (2020). Comprehensive Mapping of the Human Cytokine Gene Regulatory Network. Nucleic Acids Res. 48 (21), 12055–12073. 10.1093/nar/gkaa1055 33179750 PMC7708076

[B54] Sasse S. K. Altonsy M. O. Kadiyala V. Cao G. Panettieri R. A. Gerber A. N. (2016). Glucocorticoid and TNF Signaling Converge at A20 (TNFAIP3) to Repress Airway Smooth Muscle Cytokine Expression. Am. J. Physiology-Lung Cell Mol. Physiol. 311 (2), L421–L432. 10.1152/ajplung.00179.2016 PMC5142462 27371733

[B55] Satoh T. Nakamura S. Taga T. Matsuda T. Hirano T. Kishimoto T. (1988). Induction of Neuronal Differentiation in PC12 Cells by B-Cell Stimulatory Factor 2/interleukin 6. Mol. Cel. Biol. 8 (8), 3546–3549. 10.1128/mcb.8.8.3546 PMC363593 3264880

[B56] Singh S. Aggarwal B. B. (1995). Activation of Transcription Factor NF-Κb Is Suppressed by Curcumin (Diferuloylmethane). J. Biol. Chem. 270 (42), 24995–25000. 10.1074/jbc.270.42.24995 7559628

[B57] Skayem C. Ayoub N. (2020). Carvedilol and COVID-19: A Potential Role in Reducing Infectivity and Infection Severity of SARS-CoV-2. Am. J. Med. Sci. 360 (3), 300. 10.1016/j.amjms.2020.05.030 32631576 PMC7833103

[B58] Sokol C. L. Luster A. D. (2015). The Chemokine System in Innate Immunity. Cold Spring Harb Perspect. Biol. 7 (5). 10.1101/cshperspect.a016303 PMC4448619 25635046

[B59] Stebbing J. Sánchez Nievas G. Falcone M. Youhanna S. Richardson P. Ottaviani S. (2021). JAK Inhibition Reduces SARS-CoV-2 Liver Infectivity and Modulates Inflammatory Responses to Reduce Morbidity and Mortality. Sci. Adv. 7 (1), eabe4724. 10.1126/sciadv.abe4724 33187978 PMC7775747

[B60] Sun W. Sanderson P. E. Zheng W. (2016). Drug Combination Therapy Increases Successful Drug Repositioning. Drug Discov. Today 21 (7), 1189–1195. 10.1016/j.drudis.2016.05.015 27240777 PMC4907866

[B61] Tamura T. Yanai H. Savitsky D. Taniguchi T. (2008). The IRF Family Transcription Factors in Immunity and Oncogenesis. Annu. Rev. Immunol. 26, 535–584. 10.1146/annurev.immunol.26.021607.090400 18303999

[B62] van de Sand L. Bormann M. Alt M. Schipper L. Silke Heilingloh C. Steinmann E. (2021). Glycyrrhizin Effectively Inhibits SARS-CoV-2 Replication by Inhibiting the Viral Main Protease. Viruses 13 (4), 609. 33918301 10.3390/v13040609 PMC8066091

[B63] Walmsley S. R. Print C. Farahi N. Peyssonnaux C. Johnson R. S. Cramer T. (2005). Hypoxia-induced Neutrophil Survival Is Mediated by HIF-1α-dependent NF-Κb Activity. J. Exp. Med. 201 (1), 105–115. 10.1084/jem.20040624 15630139 PMC2212759

[B64] Wishart D. S. Feunang Y. D. Guo A. C. Lo E. J. Marcu A. Grant J. R. (2018). DrugBank 5.0: a Major Update to the DrugBank Database for 2018. Nucleic Acids Res. 46 (D1), D1074–D1082. 10.1093/nar/gkx1037 29126136 PMC5753335

[B65] Wolock S. L. Lopez R. Klein A. M. (2019). Scrublet: Computational Identification of Cell Doublets in Single-Cell Transcriptomic Data. Cel Syst. 8 (4), 281–291. 10.1016/j.cels.2018.11.005 PMC6625319 30954476

[B2] Zarubin A. Stepanov V. Markov A. Kolesnikov N. Marusin A. Khitrinskaya I. (2020). Structural Variability, Expression Profile, and Pharmacogenetic Properties of TMPRSS2 Gene as a Potential Target for COVID-19 Therapy. Genes 12 (1), 19. 10.3390/genes12010019 PMC7823984 33375616

[B66] Zhang Q. Bastard P. Liu Z. Le Pen J. Moncada-Velez M. Chen J. (2020). Inborn Errors of Type I IFN Immunity in Patients with Life-Threatening COVID-19. Science 370 (6515). 10.1126/science.abd4570 PMC7857407 32972995

[B67] Zhou Z. Ren L. Zhang L. Zhong J. Xiao Y. Jia Z. (2020). Heightened Innate Immune Responses in the Respiratory Tract of COVID-19 Patients. Cell Host & Microbe 27 (6), 883–890. 10.1016/j.chom.2020.04.017 e882 32407669 PMC7196896

