# Supplementary figures and images for: Therapeutic Targeting of Transcription Factors to Control the Cytokine Release Syndrome in COVID-19
Source: Front Pharmacol. 2021 Jun 7;12:673485. doi: 10.3389/fphar.2021.673485 (PMC8215608; doi:10.3389/fphar.2021.673485)

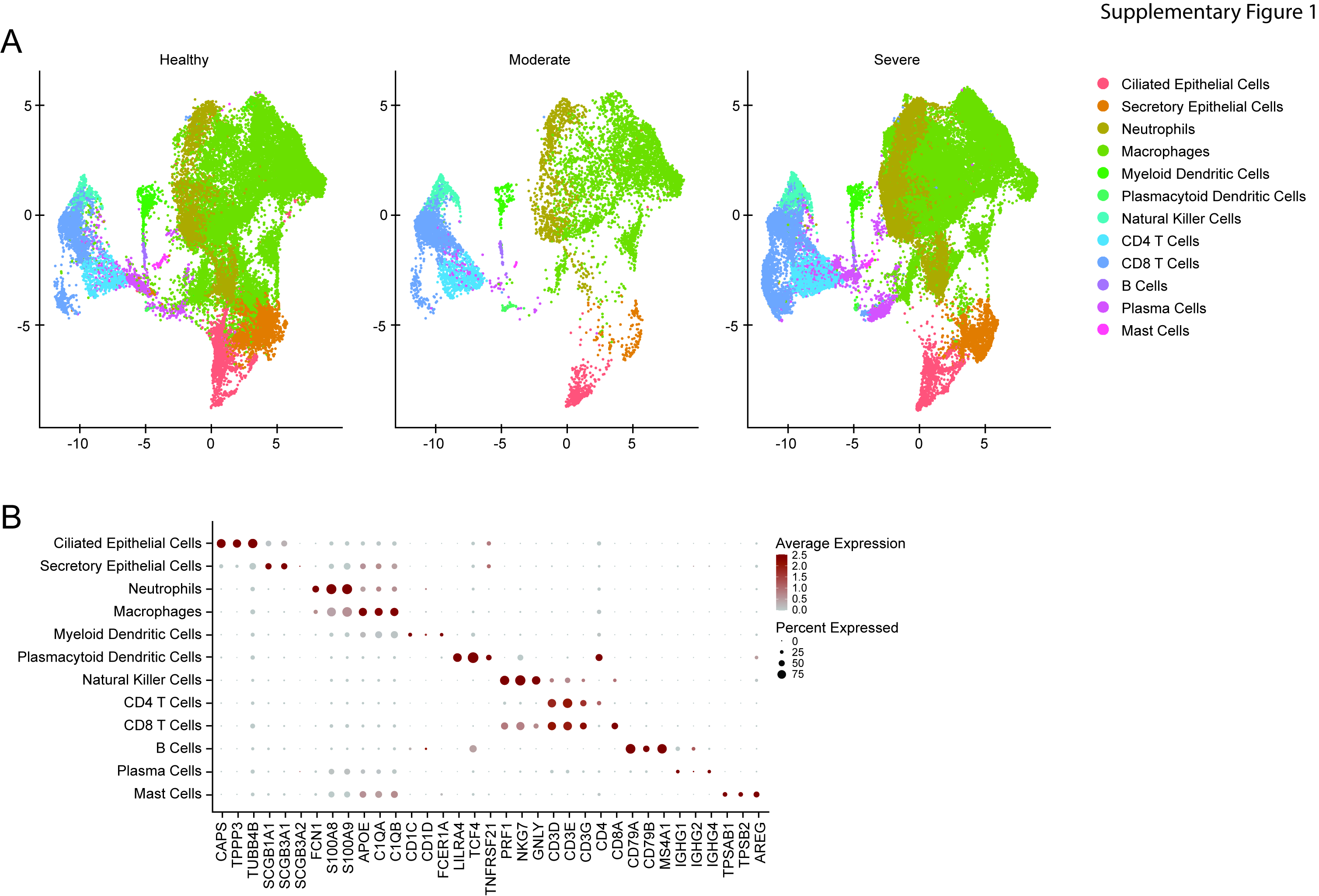

Supplement: Supplementary file 2 [file Image1.TIF]
