# Supplementary material for: Therapeutic Targeting of Transcription Factors to Control the Cytokine Release Syndrome in COVID-19
Source: Front Pharmacol. 2021 Jun 7;12:673485. doi: 10.3389/fphar.2021.673485 (PMC8215608; doi:10.3389/fphar.2021.673485)
Supplement: Supplementary file 3 [file Table3.DOCX]

| Target | Drugs |
| --- | --- |
| AHR | Atorvastatin, Diosmin, Flutamide, Ginseng, Leflunomide, Omeprazole |
| ATF2 | Pseudoephedrine |
| ATF3 | Pseudoephedrine |
| ATF4 | Pseudoephedrine |
| ATF7 | Pseudoephedrine |
| CREB1 | Adenosine phosphate, Naloxone |
| FOS | Nadroparin, Pseudoephedrine, T5224 |
| HIF1A | Carvedilol, Hydralazine |
| JUN | Irbesartan, Pseudoephedrine, T5224 |
| MYC | Acetylsalicylic acid (Aspirin), Nadroparin, |
| NFKB1 | Donepezil, Glycyrrhizic acid, Pseudoephedrine, Triflusal |
| NFKB2 | Donepezil, Glucosamine, Glycyrrhizic acid, |
| NR3C1 | Betamethasone, Budesonide, Dexamethasone, Fluticasone, Hydrocortisone, Mometasone, Methylprednisolone, Prednisolone, Prednisone, Triamcinolone |
| NR4A3 | Dasatinib |
| RELA | Dimethyl fumarate |
| RORA | Cholesterol |
| RXRA | Acitretin, Alitretinoin, Alpha-Linolenic acid, Doconexent, Etodolac, Isotretinoin, Oleic acid, Rosiglitazone |
| STAT5B | Dasatinib |
| THRB | Levothyroxine, Liothyronine, Liotrix |

*Data collected from DrugBank (<https://go.drugbank.com/>)
